# CANABIC: CANnabis and Adolescents: effect of a Brief Intervention on their Consumption – study protocol for a randomized controlled trial

**DOI:** 10.1186/1745-6215-15-40

**Published:** 2014-01-30

**Authors:** Catherine Laporte, Hélène Vaillant-Roussel, Bruno Pereira, Olivier Blanc, Gilles Tanguy, Paul Frappé, David Costa, Yoann Gaboreau, Mélanie Badin, Laurent Marty, Gilles Clément, Claude Dubray, Bruno Falissard, Pierre-Michel Llorca, Philippe Vorilhon

**Affiliations:** 1 Department of General Medicine, Faculty of Medicine of Clermont-Ferrand, 28 Place Henri Dunant, 63000 Clermont-Ferrand, France; 2 EA 7280 NPsy-Sydo, University of Auvergne, Faculty of Medicine of Clermont-Ferrand, 28 Place Henri Dunant, 63000 Clermont-Ferrand, France; 3 Office for Clinical Research and Innovation, Clermont-Ferrand University Hospital, 58 Rue Montalembert, 63000 Clermont-Ferrand, France; 4 Adult Psychiatry Department – B, Clermont-Ferrand University Hospital, 58 Rue Montalembert, 63000 Clermont-Ferrand, France; 5 Department of General Medicine, Saint-Etienne Faculty of Medicine, 15 Rue Ambroise Paré, 42023 St Etienne Cedex 2, France; 6 Department of General Medicine, Montpellier Faculty of Medicine, 2 Rue de l'École de Médecine, 34000 Montpellier, France; 7 Department of General Medicine, Grenoble Faculty of Medicine, Domaine de la Merci, BP 170 La Tronche, 38042 Grenoble Cedex 9, France; 8 Health anthropologist, 7 rue de l'église, 63450 St Amant Tallende, France; 9 Clinical Investigation Centre (Inserm CIC -501), Clermont-Ferrand University Hospital, 58 Rue Montalembert, 63000 Clermont-Ferrand, France; 10 Faculty of Medicine, Paris South, INSERM 669 Unit, Maison de Solenn, 97 Bld de Port Royal, 75679 Paris cedex 14, France; 11 EA 4681PEPRADE, University of Auvergne, Faculty of Medicine of Clermont-Ferrand, 28 Place Henri Dunant, 63000 Clermont-Ferrand, France

**Keywords:** Adolescents and young adults, Brief intervention, Cannabis, General medicine

## Abstract

**Background:**

Cannabis is the most consumed illegal substance in France. General practitioners (GPs) are the health professionals who are most consulted by adolescents. Brief intervention (BI) is a promising care initiative for the consumption of cannabis, and could be a tool for GPs in caring for adolescents who consume cannabis. The aim of the CANABIC study is to measure the impact of a BI carried out by a GP on the consumption of cannabis by adolescents of 15 to 25 years of age.

**Methods:**

A randomized clustered controlled trial, stratified over three areas (Auvergne, Languedoc-Roussillon, and Rhône - Alpes), comparing an intervention group, which carries out the BI in consultation, and a control group, which ensures routine medical care. The main assessment criterion is the consumption of cannabis by amount of joints per month, at 12 months. The amount necessary to highlight a significant difference between the two groups of 30% of consumption at 12 months is 250 patients (50 GPs, 5 patients per GP; risk α = 5%; power = 90%; intra-cluster correlation coefficient ρ = 0.2; Hawthorne effect = 15%; lost to follow-up rates for GPs = 10% and for patients = 20%). This plan is replicated for the three areas, and therefore a total of 750 patients are expected.

The secondary criteria for judgment are the associated consumption of tobacco and alcohol, the perception of the consequences of consumption, and the driving of a vehicle following consumption.

**Discussion:**

Research about BI for young cannabis users is underway. The aim of the CANABIC study is to validate a BI suited to adolescents who consume cannabis, which may be performed in the general practice. This would provide a tool for their treatment by a GP, which could be widely distributed during initial or further medical training.

**Trial registration:**

CANABIC is a randomized clustered trial (NCT01433692, registered 2011 Sept 12), PHRC funded: Clinical Research Hospital Program (Governmental Fund, Health Ministry). Date first patient randomized: March 2012.

## Background

In Europe, 12.1% of people aged from 15 to 34 state that they have consumed cannabis in the past year, vs. 16.7% in France and 24.1% for the United States [1]. In France, cannabis is the most consumed illegal substance [2]; 39% of 15 to 16-year-olds [3] and 41.5% of 17-year-olds [4] have previously smoked cannabis. This consumption relates mainly to persons below 25 years of age with an average age of experimentation of 15 [2]. Various levels of consumption are described: recent users (used at least once in the previous month), occasional (1 to 9 times per year), repeated (10 uses/year to 10 uses/month), regular (10 to 29 per month), and daily (at least once per day) [1]. In France, in 2011, 24% of young people between 15 and 16 years of age and 25% of 17-year-olds were recent consumers vs. 15% and 25% in 2007 [3,4].

Current data are clear about the risks of cannabis use: social and psychiatric risks, and highway accidents after smoking [5]. There is no consensus over the treatment of adolescents who consume cannabis. In 2008, a review of the literature explored the out-patient strategies used: motivational interviewing (MI) seems to have positive results in terms of the reduction of consumption of cannabis by young people of less than 18 years of age [6]. This was confirmed in 2013; cognitive-behavioural therapies, MI, and family therapy enable a reduction in the consumption of cannabis [7]. MI was described by Miller and Rollnick in the early 1980’s [8], as a method of interaction centred on the patient intended to modify behaviours by working on the ambivalence of the patient, naturally generated by the prospect of change. The period of psychological and physical development of adolescence makes it a target that is particularly suited to this technique. Brief intervention (BI) is a technique for motivational counselling characterized by its short duration. Its criteria of effectiveness are described using the acronym FRAMES [9]: Feedback, Responsibility, Advice, Menu, Empathy, Self-efficacy. In various European countries, trials have shown an effectiveness of BI on the consumption of alcohol [10,11]. A Canadian and an Australian study have shown the acceptability of BI among consumers of cannabis [12,13], and a trend towards a reduction in their consumption of cannabis [12,13]. A trial showed a reduction in the frequency of consumption at 3 months among young consumers who had been given a BI [14]. However, these studies were not undertaken in general medicine, but in facilities specialized in addiction or in school groups. A Swiss study showed good acceptability and feasibility of BI among general practitioners (GPs) and their young patients [15], without demonstrating its effectiveness.

The 2008 report by the World Organization of Family Doctors on mental health [16] stressed that the treatment of addiction in primary healthcare is beneficial to the improvement of the overall health condition of patients and reduces the social and economic costs that are borne by patients and their carers. In France, the structure of primary healthcare is primarily based on GPs, who are the health professionals that are most consulted by adolescents. One in seven patients consulting general medicine is aged between 11 and 20 [17]. Furthermore, for every seven patients, a GP sees one recent consumer during consultation [18]. Recourse to the healthcare system increases alongside increased cannabis consumption [19].

The 2009–2012 European Union Action Plan on drugs [20] and the 2nd French Governmental Plan (2009–2011) [21] of the Inter-ministerial Mission for the Fight Against Drugs and Drug-Addiction prioritised prevention and the development of basic and clinical research. The training of primary healthcare doctors and other healthcare professionals for early identification was also a priority area.

However, a French study in 2011 showed that only 8% of GPs questioned adolescents on their consumption of cannabis [22], indicating that there are difficulties on the part of adolescents but also on the part of GPs in addressing this topic. In 2009, the authors carried out a qualitative survey intended to identify the barriers discouraging GPs from speaking about cannabis with young patients. The majority of the 24 GPs, grouped into three focus groups, did not speak about it because they felt insufficiently trained in the identification and the treatment of cannabis consumers, and did not know how to address the topic. They were even less at ease if they had known the adolescent for a long time. The illegal aspect of the substance was an additional barrier to discussion. They all lamented the trivialisation of consumption but also deemed that it was within the remit of the private life of a young patient.

The authors also undertook a qualitative study focusing on the reluctance of adolescents to discuss their health issues with their GP, and especially their consumption of cannabis [23]. They were ambiguous with their GP, who they perceived to be both moralizing and the person who is responsible for addressing the topic. They were more comfortable speaking about it if they had known the GP for a long time and if they were alone during the consultation.

The authors hypothesize that in France, a BI in general medicine could enable identification of cannabis consumption in adolescents and propose early treatment, intended to reduce their consumption.

The main objective of the CANABIC study is to measure the effect at 12 months of a BI by GPs on cannabis consumption among recent user adolescents of 15 to 25 years of age. The secondary objectives relate to the variations in intermediate cannabis consumption at 3 and 6 months, variation in consumption associated with other methods of consumption (e.g., “bongs”), variations in consumption associated with alcohol and tobacco, the change in the perception of adolescents regarding the effects of their cannabis consumption on their personal, social, and professional lives, and on driving following the consumption of cannabis.

## Methods

The main assessment criterion is the amount of joints consumed per month at the 12^th^ month following the inclusion consultation. In the absence of consensus on the definition of a threshold for dangerous cannabis consumption, it is impossible to refer to a target level of consumption. In the light of epidemiological data (proportions of recent, repeated, regular, and daily users in the age group 15–25 years) [1,2], we considered that cannabis users consumed an average of 15 joints per month. Our working hypothesis is that the intervention will result in a 30% reduction in the stated consumption of cannabis at 12 months (that is to say five joints per month). The Hawthorne effect [24] was taken into account by estimating that adolescents of the control group would reduce their consumption by 15% (i.e., two joints per month).

A difference of 30% between the intervention group (i.e., three joints per month) and the control group appears to be clinically relevant.

Some secondary hypotheses are reported in case report forms (CRFs) at 3, 6, and 12 months by the GP: the change to other methods of cannabis consumption such as consumption by “bong” or “water pipe” and the impact of the change in cannabis use on tobacco and alcohol consumption.

Other secondary hypotheses are collected by a declaratory self-administered questionnaire completed by the patient at the start and end of the study: how it is consumed (alone or with friends, weekday or weekend, joints or bongs), changing perceptions of the danger of consumption for health, and driving after smoking.

### Study design

**Figure 1:**
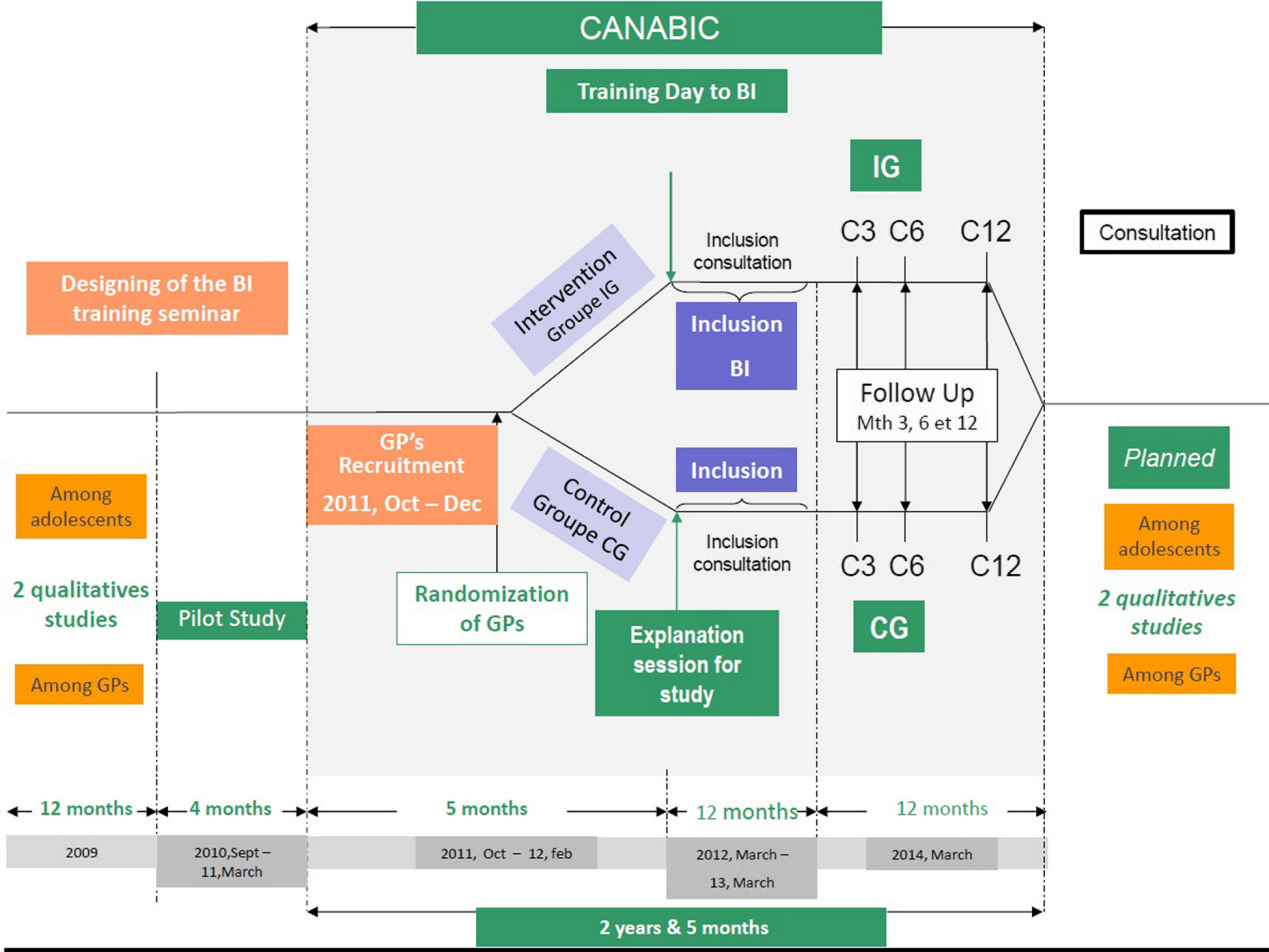
Global design of the CANABIC study.

It is a clustered randomized comparative multicentre trial, stratified by region (Auvergne, Rhône-Alps, Languedoc-Roussillon), conducted by 150 GPs, assigned to an intervention group (IG) or a control group (CG) (Figure 1). The intervention consists of a BI carried out in general medicine. The patients are included during a consultation, regardless of the reason for the consultation, and are monitored over 12 months. Each doctor must include 5 patients, that is to say 750 patients in total; 50 doctors are recruited in the three regions, 25 for the intervention group and 25 for the control group.

In the three regions, all practising GPs are invited to take part in the trial by mail. Doctors with an exclusive specialty (e.g., acupuncture, homeopathy) and doctors who have received specialized training in addiction treatment (e.g., university degree, qualification, university course) cannot take part in the trial.

Six coordinating doctors manage a group in each region. Their role is to make investigators continue inclusions, answer their questions, and link investigators on the ground to the main investigator.

### Intervention versus usual care

#### Usual care

Investigators registered in the control group provide care according to their usual practices.

#### Intervention

The investigators registered in the intervention group conduct an interview according to the BI model, defined as six key stages using the acronym FRAMES [9]:

•Feedback: returning the trial results to the patient.

•Responsibility: empower the patient by letting him establish his program for reducing or stopping consumption.

•Advice: advising moderation.

•Menu: discussing with the patient possible changes to his/her consumption.

•Empathy: treating the patient with kindness.

•Self-efficacy: allow the patient to be in charge of his/her own changes.

It is an interview outline; as this is a pragmatic trial aiming to assess the impact of BI in daily clinical practice, the delivery of the intervention is allowed to vary between health care providers. The health care providers have the flexibility to adapt the BI according to the patients’ needs.

The GPs of the intervention group are trained to carry out the BI during a standard consultation through a training day designed by the authors.

The training course was defined according to three objectives:

•Updating the knowledge of GPs regarding cannabis: levels of consumption, method of consumption, harmful effects, possibility of treatment.

•Removing communication barriers in order to facilitate discussion by returning the summary of results of the two qualitative studies. Emphasis was placed on contradictory beliefs, the expectations of adolescents, and the reluctance of doctors.

•Training GPs in BI (theoretical explanation, exercise in formulation of open-ended questions, practice through role-playing).

### Inclusion and non-inclusion criteria

#### Inclusion criteria

All young persons from 15 to 25 years of age consuming at least one joint per month for at least one year and registered with a social security scheme are eligible for the study (as stated in the law: Code of Public Health. Article L1121_11*).*

#### Non-inclusion criteria

•A patient suffering from psychiatric illness and who requires immediate specific care, at the discretion of the attending doctor.

•A patient suffering from intellectual disabilities, deafness, or who does not have a command of the French language.

•A patient previously treated for withdrawal from cannabis addiction or addiction to another substance.

•Patients who have participated in one of the preliminary qualitative studies.

### Recruitment

In both groups, the investigators identify eligible adolescents who consume cannabis by asking them the following questions: “Do you smoke cannabis? For how long have you smoked it? How much do you smoke?” The volunteer GPs recruit the first five eligible adolescents that they consult, regardless of the reason for their consultation. Participation requires a prior consent, a guarantee of anonymity, and an unconditional right to withdraw.

During this consultation, the investigators of the intervention group conduct an interview using the BI technique with the eligible and voluntary adolescents.

The doctors of the two groups provide the patient with an anonymous self-administered questionnaire to complete during the consultation. This self-administered questionnaire makes it possible to collect additional information such as method and times of consumption, monthly cost of consumption, and a cannabis abuse screening trial (CAST) score. The self-administered questionnaire is used in order to not influence the course of the consultation with adolescents of the control group and reduce the Hawthorne effect.

During the follow-up consultation at 3 and 6 months, the main assessment criterion is specified as well as cigarette and alcohol consumption. The investigators of the intervention group carry out a BI at each consultation. During the final consultation at 12 months, the doctors of the two groups provide another self-administered questionnaire.

If the adolescent does not attend the following consultation, the doctor can contact him/her by phone to make another appointment or at least to obtain the required data. To help investigators, the coordinators send a schedule of the consultations expected for each patient, as well as reminders to the GPs.

### Randomization

The doctor’s surgery is the unit of randomization. Patients of an investigator GP are assigned to the same group as him/her. Investigators from the same doctor’s surgery are randomized within the same group in order to avoid contamination bias. As the study is being carried out in three regions in which behaviours in terms of cannabis consumption and sociodemographic parameters may differ, the randomization is stratified by region and by state and is carried out using Stata, version 12 (StataCorp, College Station, USA). Taking into account the risk of contamination generated by adolescents who discuss the study with their peers while their doctors belong to different groups, collection of the name of the school/college and the class is planned in order to evaluate the possible impact of interpenetration. The same will apply for registration with one or several associations (patients were asked about their membership in a sports or cultural association; data was collected by GP during consultation).

### Statistical considerations

#### Sample size estimation

The calculation of the necessary amount of adolescents was carried out on the basis of a variation in consumption between the randomization groups of the amount of joints at 1 year, based on data from literature relating to the effect of a BI on alcohol consumption [10]. Given that the published data has heterogeneous values in terms of standard deviation associated with the quantity of joints consumed and considering the importance of a possible declarative bias, various simulations were carried out according to the value of this standard deviation (s = 1.5, 2, 3, 4 and 5) for 90% power and 5% two-sided type 1 error taking into account inclusion of five patients per GP, clustering by practice (intra-cluster correlation considered between 0.05 to 0.20 [25,26], a 10% dropout rate of GP and 20% of patients [27]). By considering the results of these simulations, a minimum of 250 patients is required to highlight a relative variation in the reduction in consumption of the amount of joints of 50% between the two groups (30% vs. 15%). This represents 50 investigators (25 in each randomization group). This design is respected in each region for an analysis stratified by region and thus highlights a variation per region. In total, 150 investigators (50 per region) must include 750 patients.

#### Statistical analyses

All data will be entered into a customized Access database (Microsoft, Redmond, Wash, USA). Analyses will be performed using Stata, version 12. All data will be analyzed by intention to treat. The tests were two-sided, with a type I error set at α = 0.05 (except for multiple comparisons when appropriate). Baseline characteristics (GPs and their patients) will be presented as the mean ± standard deviation (SD) or the median (interquartile range) for each randomization group for continuous data and as the number of patients and associated percentages for categorical parameters. Hierarchical linear regression models (mixed models) with levels for practice, individuals within practices, and repeated measurements per individual have been used to estimate effects of the intervention on number of joints smoked per month post-baseline time points. These models (intercept and slope as random effects) included an interaction between randomization group and time point, and adjusted for number of joints smoked per month at baseline, age of first consumption, gender, CAST at baseline, socioeconomic status, and GP’s characteristics. Intraclass correlation coefficients are presented by arm. The secondary analyses will compare changes on between groups with random effects models: method of consumption, supply method, perception of the effects on health, professional life, social life, and driving under the influence of alcohol will be also compared. This data will be collected through a self-administered questionnaire that the patient will complete and leave at the GP’s surgery. To assess the problem caused by missing data (GP and patients), estimation methods developed by Verbeke and Molenberg are proposed.

### Ethical considerations

The protocol received the approval of the Comité de Protection des Personnes (Commission for Public Safety) SUD-EST VI of Clermont-Ferrand on March 5th, 2010. The study is conducted in compliance with the regulations on patient confidentiality (Comité consultatif sur le traitement de l’information en matière de recherche dans le domaine de la Santé (Advisory Committee on Data Processing for Matters of Research in the Field of Healthcare) and Commission Nationale de l’Information et des Libertés (National Commission for Data Protection) agreements). Inclusion is voluntary and subject to strict anonymity and medical confidentiality. The description of this design follows the CONSORT recommendations for reporting on trials (http://www.consort-statement.org). The trial has been registered on http://clinicaltrials.gov/: NCT01433692.

## Discussion

The main assessment criterion being consumption by amount of joints smoked in each group, it is difficult to establish a quantity of consumption for cannabis (e.g., shared joints, joints that are more or less 'concentrated’). The epidemiologic data usually quantifies consumption by 'use’ of cannabis: that is to say 'the amount of times that the adolescent has smoked’ [1,3,4]. Concordances were established; half of recent smokers smoke one or less than one joint per use, and 70% of them smoke two or less. Regular or occasional smokers smoke five joints or more per use in 30% of cases [4]. It would have been possible to assess consumption more objectively using biological sampling (hair samples, urine tests). However, early treatment in general medicine requires a relationship of trust between the doctor and the patient; mandatory biological testing would have been badly received by adolescents. The CAST score (Cannabis Abuse Screening Test) is only assessed at the start of the study, as it has the sole objective of detecting problematic use of cannabis [28,29], but is not suited to measuring the progression of consumption. It will enable comparison of the progression of consumption in adolescents who had an initial score which revealed harmful use and the others. The primary and secondary criteria are collected during the preceding month, then at 3, 6, and 12 months. Collection during the preceding month was chosen in order to limit memorization bias, while maintaining a sense of overall consumption, as certain patients do not consume cannabis regularly.

Any adolescents who consume cannabis are eligible, on the condition that they smoke at least one joint per month. These levels of consumption may appear highly dispersed, but the European School Survey Project on Alcohol and other Drugs and the Survey on Health and Consumption administered during the Defence Preparation Day data described correlations between quantity consumed and certain behaviours (without causal links to date), e.g., runaways, school absenteeism, depression, and acts of violence. The difference in behaviour between occasional and regular consumers is less significant than between occasional consumers and non-consumers [18]. These consumers thus constitute a homogeneous group as regards behaviour. The selection of the 15–25 age bracket appears to be relevant for carrying out a trial on intervention on cannabis consumption. Fifteen years of age is the average age of experimentation with cannabis, and 25 years of age is the apex of consumption [2]. Consumption is quite homogeneous; in the 19–25 age bracket, 14.6% are repeat consumers [2], and in 12–19 year olds, 16.4% are repeat consumers [3].

The participation of GPs in a study depends mainly on the applicability of the topic to their practice, remuneration, the support of the study coordinators, and the feedback of information [30]. Lack of time and the administrative burden are the main obstacles to the inclusion and follow-up of patients by the GP [31]. The illegal aspect of cannabis consumption in France can also present an obstacle to the recruitment of GP investigators and the inclusion of adolescents. The Scientific Committee of CANABIC developed a strategy relating to doctors and patients in order to optimize inclusion [25-31]: GPs are remunerated, the CRFs can be completed quickly and reattempts are carried out by the regional coordinators. A pilot study tested the feasibility of the study among volunteer GPs. Analysis of the difficulties encountered by the investigators made it possible to optimize certain features of the protocol through extension of the inclusion period (12 months) and the absence of any specific qualification in addiction treatment of the investigators. It does indeed appear that when the practitioners have such a qualification, they already apply these specific interview techniques and the training day has little impact on them. In order to strengthen the participation of patients, they were sensitized to the danger of cannabis to their own health and were empowered, through the various stages of the BI [25].

There is currently broad consensus deeming that it is important to identify at an early stage potentially addictive behaviours in adolescents, as on the one hand progression towards cannabis dependency partly depends on the age at which consumption begins [32], and on the other primary prevention actions have not, to date, demonstrated the effectiveness expected [33]. In several studies, the BI appears to be a relevant tool in terms of acceptability and feasibility among cannabis-using adolescents [12-15]. A trial that does not demonstrate effectiveness raises the question of the homogeneity of the BI carried out [34]. Recent trials have shown promise regarding the impact at 6 months of a BI carried out for the consumption of several drugs [35,36]. Solid evidence of the effectiveness of a BI on the reduction in cannabis consumption remains to be found, particularly when it is carried out by GPs. Easily accessible through ongoing or initial training, the BI should improve the overall care provided to adolescents who consume cannabis and are monitored in primary care.

## Trial status

The CANABIC study is ongoing.

## Abbreviations

BI: Brief intervention; CAST: Cannabis abuse screening trial; CG: Control group; FRAMES: Feedback, Responsibility, Advice, Menu, Empathy, Self-efficacy; CRF: Case report form; GP: General practitioner; IG: Intervention group; MI: Motivational interviewing.

## Competing interests

The authors declare that they have no competing interests.

## Authors’ contributions

All authors took part in the design of the study. CL is responsible for operational supervision of the study as lead principal investigator and drafted the manuscript. BP participated in the study design and carried out the statistical analysis. OB was co-organizer of the training courses for investigators and is coordinator of the follow-up of the study and data capture. HVR, LM, and GT participated in the drafting of the protocol and the preliminary studies. DC, PF, YG, MB, and GT ensured regional coordination for the completion of the trial. BF participated in the statistical analysis and drafting of the manuscript. PML, CD, and GC participated in the designing of the study and are the scientific co-directors of the project. PV initiated and has scientific responsibility for the project. All authors participated in the preparation of the final manuscript and approved it for publication.

## Authors’ information

Principal Investigator; Catherine Laporte.
